# 39例肺肉瘤样癌临床病理特征及预后分析

**DOI:** 10.3779/j.issn.1009-3419.2024.101.18

**Published:** 2024-07-20

**Authors:** Cen CHEN, Zhanliang REN, Yujie DONG, Ying WANG, Yuan GAO, Hongxia LI, Tongmei ZHANG

**Affiliations:** ^1^712000 咸阳，陕西中医药大学; ^1^Shaanxi University of Chinese Medicine, Xianyang 712000, China; ^2^712000 咸阳，陕西中医药大学附属医院胸心外科; ^2^Department of Thoracic and Cardiac Surgery, Affiliated Hospital of Shaanxi University of Chinese Medicine, Xianyang 712000, China; ^3^101149 北京，首都医科大学附属北京胸科医院病理科; ^3^Department of Pathology, Beijing Chest Hospital, Capital Medical University, Beijing 101149, China; ^4^101149 北京，首都医科大学附属北京胸科医院肿瘤科; ^4^Department of Medical Oncology, Beijing Chest Hospital, Capital Medical University, Beijing 101149, China; ^5^101149 北京，首都医科大学基础临床联合实验室; ^5^Laboratory for Clinical Medicine, Capital Medical University, Beijing 101149, China

**Keywords:** 肺肉瘤样癌, 临床特征, 病理特征, 预后, Pulmonary sarcomatoid carcinoma, Clinical characteristics, Pathological characteristics, Prognosis

## Abstract

**背景与目的:**

肺肉瘤样癌（pulmonary sarcomatoid carcinoma, PSC）是非小细胞肺癌（non-small cell lung cancer, NSCLC）的罕见类型，具有低发病率、高度恶性、强侵袭性、预后差的特点，当前无标准治疗方案。本研究拟通过收集PSC患者临床病理特征、当前诊治情况并分析预后因素，总结诊治经验，旨在提高临床对PSC的认识。

**方法:**

回顾性收集2013年12月至2023年12月于北京胸科医院确诊、接受治疗且临床资料完整的39例PSC患者的人口学信息、临床病理特征、肿瘤原发灶-淋巴结-转移（tumor-node-metastasis, TNM）分期和诊疗方案资料，并完成临床预后随访。应用Kaplan-Meier法进行单因素生存分析。

**结果:**

39例PSC患者年龄范围45-76岁，其中男性35例，女性4例，首诊临床表现缺乏特异性；20例患者接受手术治疗，19例患者行姑息性放化疗或对症支持治疗。患者1、5年生存率分别为61.90%、35.20%。单因素分析结果提示恶性肿瘤家族史、肿瘤部位、TNM分期、淋巴结转移、远处转移、是否手术、手术类型、治疗方案、细胞程序性死亡配体1（programmed cell death ligand 1, PD-L1）蛋白表达≥1%、间质上皮细胞转化因子（mesenchymal-epithelial transition factor, MET）通路异常与患者总生存期（overall survival, OS）有关（P<0.05）；多因素分析结果显示，淋巴结转移是患者OS的独立影响因素（P=0.037）。

**结论:**

PSC临床发病率低，多见于有吸烟史的老年男性。PD-L1蛋白表达≥1%及MET通路异常可提示患者不良预后，淋巴结转移是患者OS的独立危险因素。以手术为主的综合治疗是早期及局部晚期患者的主要治疗模式，靶向治疗和免疫治疗的临床应用价值有待进一步探索。

## Abstract (en)

**Background and objective:**

Pulmonary sarcomatoid carcinoma (PSC) is a rare subtype of non-small cell lung cancer (NSCLC), which is featured by low incidence, high malignancy rate, robust aggressive behavior and inferior prognosis. To date, there is no standardized treatment. The aim of this study is to better understand and accumulate more clinical experience of the disease by summarizing the clinicopathological features, diagnosis methods, therapeutic regimen and prognostic factors of PSC.

**Methods:**

A total of 39 patients with PSC who diagnosed and received treatment in Beijing Chest Hospital from December 2013 to December 2023 were retrospectively recruited, and information including demographic characteristics, clinicopathological features, tumor-node-metastasis (TNM) stage, diagnosis method and therapeutic regimen were carefully collected. Meanwhile, follow-up was conducted. Kaplan-Meier method was used to analyze the prognostic factors of the disease.

**Results:**

The PSC patients in this study ranged in age from 45 to 76 years old, including 35 males and 4 females. There were no specific clinical manifestations of PSC at initial diagnosis. Among the 39 patients, 20 underwent surgical resection and 19 received palliative chemoradiation or symptomatic supportive treatment. The 1-year and 5-year survival rates were 61.90% and 35.20% respectively. Univariate analysis indicated that family history of carcinoma, primary tumor site, TNM stage, lymph node metastasis, distant metastasis, whether or not received surgical resection, surgical method, treatment regimens, tumor tissue programmed cell death ligand 1 (PD-L1) expression ≥1% and mesenchymal-epithelial transition factor (MET) pathway abnormalities were correlated with the overall survival (OS) of patients (P<0.05). In the subsequent multivariate analysis, lymph node metastasis emerged as the only independent prognosticator in predicting inferior OS (P=0.037).

**Conclusion:**

PSC is rarely seen in clinical practice and commonly occurs in elder men with smoking history. Tumor tissue PD-L1 expression ≥1% and MET abnormalities may predict inferior prognosis of PSC and lymph node metastasis was determined as the independent prognosticator of PSC. Surgical resection along with adjuvant medical treatment is the cornerstone for early and locally advanced patients, and the clinical utility of molecular targeting therapy and immunotherapy in PSC needs to be further investigated.

肺肉瘤样癌（pulmonary sarcomatoid carcinoma, PSC）是一种罕见的非小细胞肺癌（non-small cell lung cancer, NSCLC），约占所有肺癌的0.5%^[1]^，侵袭性强，临床预后差。世界卫生组织（World Health Organization, WHO）将PSC分为多形性癌、梭形细胞癌、巨细胞癌、癌肉瘤和肺母细胞瘤五大类病理类型^[2]^。因其发病率较低，目前相关研究较少，临床无标准诊疗方案。总结临床患者的临床病理特征及诊治和预后信息，积累经验，有助于提高PSC的临床诊治水平。本研究回顾性分析本院39例PSC患者的临床病理资料，结合文献分析其诊治方式和预后因素，旨在提高对PSC的认识。

## 1 资料与方法

### 1.1 患者资料

研究对象为2013年12月至2023年12月于首都医科大学附属北京胸科医院经病理学诊断并接受治疗且随访资料完整的39例患者。纳入标准：符合WHO（2004年版）中PSC的诊断标准；年龄>18周岁；患者病史及随访资料信息完整性好。排除标准：合并影响肿瘤治疗的重要脏器功能障碍；患有精神疾病或存在认知障碍者；既往诊断其他恶性肿瘤病史者。收集患者的人口学信息、吸烟史、首诊临床表现、原发肿瘤大小、肿瘤分级、国际通用肿瘤分期系统肿瘤原发灶-淋巴结-转移（tumor-node-metastasis, TNM）分期、病理免疫组化结果、诊疗方案和预后信息，其中肿瘤分级、TNM分期采用国际抗癌联盟（Union for International Cancer Control, UICC）分期标准（2010年第7版）。本研究涉及的患者及组织标本来自首都医科大学附属北京胸科医院肺癌队列，获得首都医科大学附属北京胸科医院伦理委员会的审核批准（No.LW-2024-014），并获得患者或家属的知情同意。

### 1.2 病理检查

本研究中所有标本均行苏木精-伊红（hematoxylin-eosin, HE）染色形态学观察及免疫组化检查，符合2004年版WHO肺、胸膜、胸腺和心脏肿瘤组织学分类标准中PSC的病理诊断标准。主要试剂包括广谱型细胞角蛋白（cytokeratin pan, CKpan）抗体试剂（福州迈新生物技术开发有限公司，货号：Kit-0009），Vimentin抗体试剂（Roche公司，货号：K20721）。

### 1.3 随访

采用首都医科大学附属北京胸科医院病案管理系统，通过病历查阅、互联网复诊和电话等方式对患者进行随访。随访时间为自确诊之日至末次随访日期或死亡时间；总生存期（overall survival, OS）为自确诊时间至死亡时间或末次随访时间。末次随访时间为2023年12月20日，其中18例（46.15%）患者存活，21例（53.85%）患者死亡，中位随访时间30.27个月。

### 1.4 统计学方法

采用SPSS 26.0统计软件进行数据分析，Kaplan-Meier法进行生存分析并使用GraphPad Prism 9.5软件绘制生存曲线，Log-rank检验进行显著性检验，Cox风险回归模型对预后相关因素进行分析，P<0.05为差异有统计学意义。

## 2 结果

### 2.1 临床特征

本研究患者中男性35例，女性4例，男女比例为8.75:1。发病年龄范围45-76岁，中位年龄为63岁。30例（76.92%）患者有吸烟史，其中28例（71.79%）患者有重度吸烟史（吸烟指数≥400年支）。3例（7.69%）患者存在恶性肿瘤家族史。4例（10.26%）患者无明显症状，体检发现肺部病变，27例（69.24%）患者出现咳嗽、咳痰、咯血、发热等呼吸道症状，6例（15.38%）患者主要症状为胸背痛，1例（2.56%）患者出现乏力，1例（2.56%）患者因发现胸壁肿块就诊。回顾性分析患者首诊胸部计算机断层扫描（computed tomography, CT）影像学表现，有43.59%（17例）的患者原发部位位于左肺，56.41%（22例）患者的原发肿瘤位于右肺，其中左肺上叶14例（35.90%）、左肺下叶3例（7.69%）、右肺上叶10例（25.64%）、右肺中叶2例（5.13%）、右肺下叶8例（20.51%），原发肿瘤位于上叶的占61.54%（24例），位于中/下叶的占33.33%（13例），有2例（5.13%）患者未能明确分叶部位，均位于右肺。根据TNM分期，I和II期患者占比均为15.38%（6例），III期患者占比23.08%（9例），IV期患者数量最多，占比46.16%（18例）。其中，肿瘤最大径≤5 cm者14例（35.90%），>5 cm者25例（64.10%）。有淋巴结转移患者27例（69.23%），无淋巴结转移患者12例（30.77%），存在远处转移的患者17例（43.59%），无远处转移的患者22例（56.41%）（表1）。

**表1:** 39例PSC患者的临床特征及OS单因素预后分析

Variables	n (%)	Univariate analysis	
		HR (95%CI)	P
Gender		0.693 (0.160-2.997)	0.621
Female	4 (10.26)		
Male	35 (89.74)		
Age (yr)		1.109 (0.457-2.686)	0.819
<60	15 (38.46)		
≥60	24 (61.54)		
Smoking history		0.852 (0.312-2.331)	0.755
No	9 (23.08)		
Yes	30 (76.92)		
Smoking index		0.374 (0.082-1.694)	0.184
<400	2 (5.13)		
≥400	28 (71.79)		
Family history of malignant tumors		4.126 (1.147-14.845)	0.019
No	36 (92.31)		
Yes	3 (7.69)		
Clinical symptoms		1.319 (0.813-2.142)	0.771
None	4 (10.26)		
Respiratory symptom	27 (69.24)		
Ache	6 (15.38)		
Fatigue	1 (2.56)		
Chest wall mass	1 (2.56)		
Tumor site		1.145 (0.861-1.522)	0.020
Superior lobe of left lung	14 (35.90)		
Inferior lobe of left lung	3 (7.69)		
Superior lobe of right lung	10 (25.64)		
Middle lobe of right lung	2 (5.13)		
Inferior lobe of right lung	8 (20.51)		
NOS	2 (5.13)		
TNM stage		3.343 (1.676-6.667)	<0.001
I	6 (15.38)		
II	6 (15.38)		
III	9 (23.08)		
IV	18 (46.16)		
Maximum tumor diameter (cm)		2.617 (0.947-7.228)	0.054
≤5	14 (35.90)		
>5	25 (64.10)		
Lymphatic metastasis		3.773 (1.107-12.860)	0.023
No	12 (30.77)		
Yes	27 (69.23)		
Distant metastasis		9.539 (3.259-27.924)	<0.001
No	22 (56.41)		
Yes	17 (43.59)		
Variables	n (%)	Univariate analysis	
		HR (95%CI)	P
Surgery		0.264 (0.104-0.671)	0.011
No	19 (48.72)		
Yes	20 (51.28)		
Therapy methods		1.402 (0.881-2.228)	<0.001
Surgery alone	6 (15.38)		
Surgery combined with postoperative adjuvant therapy	14 (35.90)		
Chemotherapy or radiotherapy or targeted therapy or immunotherapy	11 (28.21)		
Treatment abandonment	5 (12.82)		
NOS	3 (7.69)		
Surgery method		3.870 (1.696-8.834)	<0.001
Pulmonary lobectomy	15 (38.46)		
Lung sleeve lobectomy	1 (2.56)		
Total pneumonectomy	4 (10.26)		
PD-L1 protein expression		0.301 (0.116-0.779)	0.009
≥1%	19 (48.72)		
<1%	20 (51.28)		
MET pathway		6.077 (0.791-46.657)	0.049
Abnormal	6 (15.38)		
Normal	33 (84.62)		

OS: overall survival; PSC: pulmonary sarcomatoid carcinoma; HR: hazard ratio; NOS: not otherwise specified; PD-L1: programmed cell death ligand 1; MET: mesenchymal epithelial transition; TNM: tumor-node-metastasis.

### 2.2 病理及免疫组化

39例患者均通过手术、肺/胸膜穿刺活检或支气管镜活检等方式进行病理学确诊，其中17例（43.59%）患者经根治性手术切除肿块及淋巴结清扫术获取标本，18例（46.15%）患者采用肺/胸膜穿刺活检取材，4例（10.26%）患者采用支气管镜活检获取组织标本，均经病理学确诊。按照WHO的病理分型，有6例（15.38%）多形性癌，8例（20.51%）梭形细胞癌，1例（2.56%）巨细胞癌，24例（61.54%）未明确具体分型（图1A）。39例患者免疫组化检测结果显示，间质细胞标志物Vimentin阳性率82.05%（32例），上皮源性标志物CKpan阳性率74.36%（29例）、角蛋白7（cytokeratin 7, CK7）阳性率38.46%（15例）、角蛋白18（cytokeratin 18, CK18）阳性率30.77%（12例）、角蛋白（cytokeratin, CK）和角蛋白5/6（cytokeratin 5/6, CK5/6）阳性率均为12.82%（5例）（图1B），上皮膜抗原（epithelial membrane antigen, EMA）阳性率17.95%（7例），Ki-67阳性率56.41%（22例），细胞程序性死亡配体1（programmed cell death ligand 1, PD-L1）蛋白表达≥1%的患者占比48.72%（19例），其中PD-L1蛋白高表达（≥50%）的患者占41.03%（16例），甲状腺转录因子1（thyroid transcription factor-1, TTF-1）阳性率为38.46%（15例），同时，有4例（10.26%）患者检测到细胞间质上皮转化因子（cellular-mesenchymal epithelial transition factor, c-MET）蛋白阳性。图2中显示PSC病理免疫组化的染色，肿瘤细胞呈梭形细胞肉瘤样改变，Vimentin及CKpan标志物表达阳性。

**图1:**
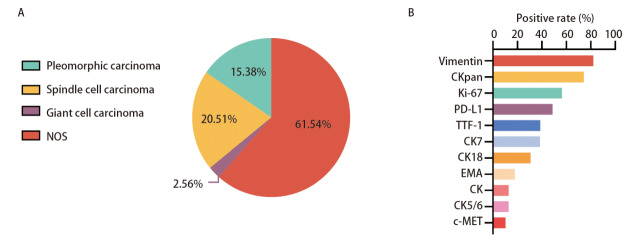
PSC患者病理分型及免疫组化分析。A：病理分型的数量比例分析；B：免疫组化标志物数量比例分析。

**图2:**
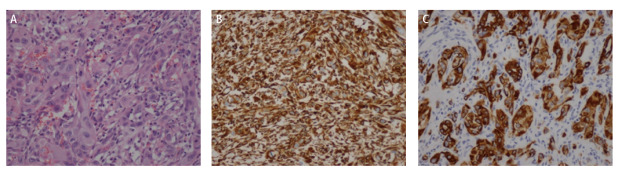
PSC病理及免疫组化。A：肿瘤细胞呈梭形细胞肉瘤样改变（HE染色，×200）；B：免疫组化标记Vimentin呈阳性表达（×200）；C：免疫组化标记CKpan呈阳性表达（×200）。

### 2.3 基因突变

39例患者中31例（79.49%）有完整基因检测信息，其中26例（66.67%）患者检测到基因突变。20例（51.28%）采用第二代测序（next-generation sequencing, NGS）检测，9例（23.08%）采用突变扩增系统（amplification refractory mutation system, ARMS）荧光定量聚合酶链式反应（polymerase chain reaction, PCR）法，2例（5.13%）行院外基因检测，具体方法不详（图3A）。其中，13例（41.94%）患者肿瘤蛋白p53（tumor protein p53, TP53）基因突变阳性，2例（6.45%）患者MET 14外显子跳跃突变，4例（12.90%）患者检测到Kirsten鼠类肉瘤病毒癌基因（Kirsten rat sarcoma viral oncogene, KRAS）基因突变，2例（6.45%）患者原癌基因转染时重排（rearranged during transfection, RET）基因突变阳性，1例（3.23%）患者表皮生长因子受体（epidermal growth factor receptor, EGFR）基因突变（图3B）。

**图3:**
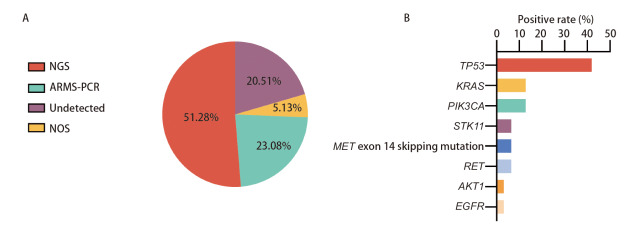
PSC患者基因突变分析。A：不同基因检测方法的数量比例对比；B：不同突变基因的数量比例对比。

### 2.4 治疗方法

39例患者中6例（15.38%）接受单纯手术治疗，14例（35.90%）接受手术联合术后辅助治疗，其中1例接受术前新辅助化疗，11例（28.21%）行化疗、放疗、靶向治疗或免疫治疗，5例（12.82%）因各种原因确诊后放弃进一步治疗，3例（7.69%）确诊后于院外治疗，信息缺失。20例手术治疗患者的外科术式包括15例（38.46%）肺叶切除术，1例（2.56%）肺叶袖式切除术，4例（10.26%）全肺切除术（表1）。

### 2.5 生存分析

39例患者的中位OS为18.20个月（图4），患者的1年生存率为61.90%，5年生存率为35.20%。单因素分析结果显示，恶性肿瘤家族史（P=0.019）、肿瘤部位（P=0.020）、TNM分期（P<0.001）、淋巴结转移（P=0.023）、远处转移（P<0.001）、是否手术（P=0.011）、手术类型（P<0.001）、治疗方案（P<0.001）、肿瘤组织PD-L1蛋白表达（P=0.009）（图5A）对PSC患者的OS有显著影响，同时，6例（15.38%）患者MET通路异常，包含c-MET蛋白阳性4例和MET基因14外显子跳跃突变2例，同样与PSC患者OS相关（P=0.049）（图5B）；而患者年龄、性别、吸烟史、临床表现与PSC患者OS无关（P>0.05）（表1）。多因素分析结果显示，淋巴结转移是患者OS的独立影响因素（P=0.037）（表2）。

**图4:**
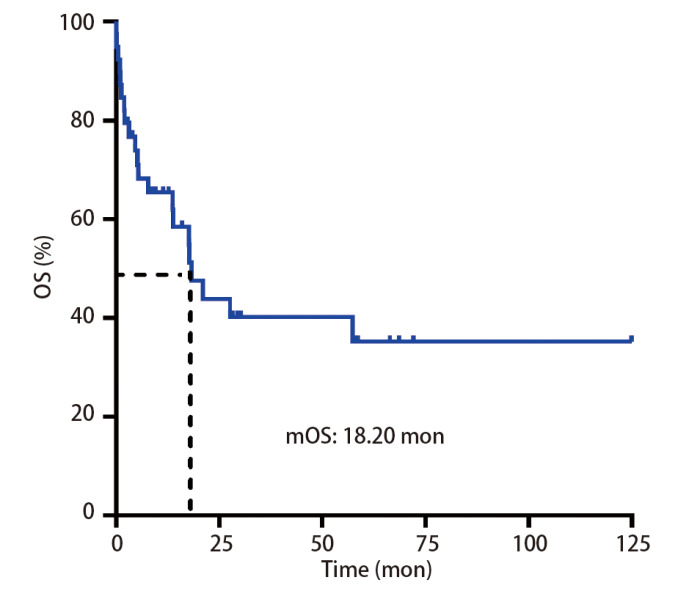
39例PSC的生存曲线

**图5:**
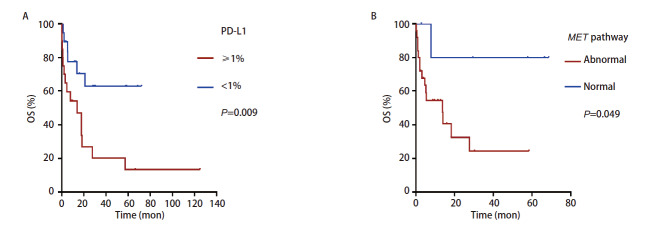
PSC患者OS的Kaplan-Meier生存分析。A：PD-L1的生存曲线；B：MET通路的生存曲线。

**表2:** 39例PSC患者OS多因素预后分析

Variables	Wald	P	HR (95%CI)
Family history of malignant tumors (Yes vs No)	1.431	0.232	3.384 (0.459-24.948)
Tumor site (Superior lobe of left lung vs inferior lobe of left lung vs superior lobe of right lung vs middle lobe of right lung vs inferior lobe of right lung vs NOS)	1.633	0.201	0.749 (0.481-1.167)
TNM stage (I-III vs IV)	1.212	0.271	2.169 (0.546-8.614)
Lymphatic metastasis (Yes vs No)	4.345	0.037	5.875 (1.111-31.055)
PD-L1 (≥1% vs <1%)	1.932	0.165	0.350 (0.080-1.538)
MET pathway (Abnormal vs Normal)	1.604	0.205	0.254 (0.030-2.119)

## 3 讨论

数据统计^[3]^显示，肺癌是我国发病率及死亡率最高的恶性肿瘤，与其他NSCLC相比较，PSC侵袭性强，恶性程度较高，发病率低，临床诊治经验不足，治疗方式有限，预后较差。

既往文献报道PSC患者多为60岁以上男性，与吸烟史密切相关^[4]^，本研究患者中位年龄63岁，男性患者为主，与文献报道一致。本研究中76.92%的患者有吸烟史，且其中超过半数具有重度吸烟史，但分析显示吸烟史与OS并无统计学意义（P=0.755），与毛玉焕等^[5]^研究结果一致。

PSC无特异性临床表现，与其他肺癌类型类似，大多数患者以呼吸道症状为首发症状，如咳嗽、咳痰、咯血、痰中带血等，早期鉴别困难。在影像学检查中，大多数患者初诊时肿块较大，文献^[6]^报道PSC肿瘤直径<5 cm的病灶较少坏死且强化均匀，而直径>5 cm的病灶常出现坏死，并出现周边强化，本研究患者肿瘤最大径在2.20-12.20 cm，中位值为5.20 cm，64.10%患者的肿瘤最大径>5 cm，多位于两肺上叶，其中左肺上叶占35.90%、左肺下叶占7.69%、右肺上叶占25.64%、右肺中叶占5.13%、右肺下叶占20.51%，与李巧珍等^[7]^报道一致。PSC侵袭性强，易发生远处转移，多数患者就诊时已处于中晚期，本研究中III和IV期患者占比69.24%，患者淋巴结转移和远处转移与OS相关，淋巴结转移是OS的独立危险因素，具有重要的预后价值，结果与杨昆等^[8]^报道一致，但不同在于后者研究对象均为手术治疗后，且认为肿瘤直径也是OS的独立影响因素。另有研究^[9]^指出尽管淋巴结转移与OS有一定相关性，但并不是OS的独立危险因子。不同研究结果间存在差异可能与纳入人群不同、整体样本量少有关，另外回顾性分析性质也在一定程度上会导致本研究结论出现偏倚。

目前研究认为，PSC肿瘤组织成分混合，与上皮间充质转化（epithelial-mesenchymal transition, EMT）密切相关^[10]^，瘤内和瘤间具有高度的异质性^[11,12]^。大多数参与EMT的蛋白，如Vimentin间质细胞标志物^[13]^，在PSC中过表达，同时如CKpan、EMA等上皮样标志物阳性表达率高，意味着细胞间黏附能力下降，较其他NSCLC表现出早期局部侵袭和远处转移的侵袭性临床特征。本研究22例Ki-67阳性表达患者中，19例患者Ki-67增殖指数>30%，提示肿瘤细胞增殖活跃^[14]^，亦能提示PSC侵袭性较强，上述标志物有助于PSC的诊断鉴别，但均与OS无显著相关。

PSC的治疗方案尚未标准化，目前治疗原则与其他NSCLC相似，首选手术治疗，辅以化疗、放疗、靶向治疗、免疫治疗等综合治疗。与既往文献报道结果一致^[9]^，本研究20例患者接受了手术治疗，19例患者行姑息性放化疗或对症支持治疗，生存分析发现是否接受手术切除以及手术术式均与患者OS相关，进一步确认了手术治疗在PSC患者中的基石地位。因多数患者初诊时已是中晚期，无手术治疗指征，除放化疗外，PSC患者能否在免疫治疗或靶向治疗中获益仍有待真实世界研究证实。

既往文献^[12,15,16]^报道PSC患者肿瘤组织多存在PD-L1蛋白表达，且研究^[17]^表明PD-L1蛋白高表达的患者在免疫治疗中疗效较好。本研究中，患者肿瘤组织PD-L1蛋白中位表达水平70%，3例患者PD-L1蛋白高表达且应用程序性死亡受体1（programmed cell death protein 1, PD-1）免疫检查点抑制剂卡瑞利珠单抗或信迪利单抗免疫联合化疗，中位OS为13.73个月，提示PSC应用免疫治疗有显著优势。本研究中以肿瘤组织PD-L1蛋白表达阳性细胞占比≥1%为阳性，<1%为阴性为标准，分析PD-L1蛋白表达的预后价值，显示PD-L1蛋白是否阳性与OS的相关性具有统计学意义，提示存在PD-L1蛋白的患者免疫应答率较高，免疫检查点抑制剂在这部分患者中的使用可有效提高生存期。但本研究的样本量较少，还需要更多来自真实世界的临床数据完善免疫治疗在PSC的应用价值。

近年来靶向治疗改变了肺癌临床实践的治疗格局，为患者带来了新的治疗希望，众多研究者也在PSC中探求靶向治疗的可能性。MET通路异常包括MET 14外显子跳跃突变、MET基因扩增和c-MET蛋白过表达，是NSCLC患者潜在获益的治疗靶点^[18,19]^。文献^[20]^报道PSC中MET 14外显子跳跃突变的发病率为4.9%-31.8%，高于其他NSCLC亚型，是NSCLC的MET靶向治疗被批准的第一个靶点^[21]^。本研究中6例患者提示MET通路异常，包括2例MET 14外显子跳跃突变及4例c-MET蛋白过表达。我们的研究发现MET通路异常与患者较长的OS有关，差异有统计学意义，与Gow等^[22]^研究结果相似。研究结论提示MET通路的改变可能影响患者生存期，有一定的预测价值，但需要更大样本量的数据支持。另外我们未发现常见基因突变如TP53、KRAS等与患者生存期相关，考虑与回顾性分析中样本量较少且既往基因检测可及性差有关。

既往很多研究对PSC临床病理特征及预后影响因素进行分析，李园园^[23]^、陈钟^[9]^等强调了早期及局部晚期患者手术切除的重要作用并提出免疫治疗及靶向治疗的巨大临床应用潜力，但未对肿瘤组织PD-L1蛋白表达及MET通路状态进行相关预后分析，本研究发现肿瘤组织PD-L1蛋白表达和MET通路异常与OS均有显著相关性，为免疫治疗和靶向治疗在PSC中的临床应用提供了一定的研究基础。需要指出的是，本研究样本量相对较少，且部分患者采用小标本进行病理诊断，可能会影响研究结论外推，后续需要纳入更多PSC患者进行分析以提供充足的循证医学证据。

综上，PSC患者多见于有吸烟史的老年男性，首诊缺乏特异性临床表现且初诊时常处于中晚期，临床预后较差且缺乏标准化治疗。以手术为主的综合治疗方式能为早期及局部晚期患者带来生存获益。对于晚期无手术机会的PSC患者，化放疗是主要治疗手段，另有部分患者可在免疫及靶向治疗中获益，需进一步扩大样本量确认。
